# Retraction Note: Astragalus saponins affect proliferation, invasion and apoptosis of gastric cancer BGC-823 cells

**DOI:** 10.1186/s13000-017-0657-9

**Published:** 2017-09-07

**Authors:** Tao Wang, Xiaoyan Xuan, Min Li, Ping Gao, Yuling Zheng, Wenqiao Zang, Guoqiang Zhao

**Affiliations:** 1 Department of Hemato-tumor, The First Affiliated Hospital of Henan University of TCM, Zhengzhou, People’s Republic of China; 2 Department of Microbiology and Immunology, College of Basic Medical Sciences, Zhengzhou University, Zhengzhou, People’s Republic of China; 3 Henan University of TCM, Zhengzhou, People’s Republic of China

## Retraction

This article has been retracted by the authors [1] because large portions of text have been duplicated from a number of previously published articles, including Tin et al., 2007 [2] and Auyeung et al., 2009 [3]. All authors agree with the retraction.
